# Spike mutations in SARS-CoV-2 AY sublineages of the Delta variant of concern: implications for the future of the pandemic

**DOI:** 10.2217/fmb-2021-0286

**Published:** 2022-02-15

**Authors:** Daniele Focosi, Fabrizio Maggi, Scott McConnell, Arturo Casadevall

**Affiliations:** ^1^North-Western Tuscany Blood Bank, Pisa University Hospital, Pisa, Italy; ^2^Department of Medicine & Surgery, University of Insubria, Varese, Italy; ^3^Laboratory of Microbiology, ASST Sette Laghi, Varese, Italy; ^4^Department of Medicine, Johns Hopkins School of Public Health & School of Medicine, Baltimore, MD 21287, USA

**Keywords:** AY, COVID-19, delta, SARS-CoV-2, spike

The ongoing COVID-19 pandemic has caused 271 million cases and 5.32 million deaths worldwide as of 15 December 2021, and represents an unprecedented opportunity to monitor the natural evolution of a human pathogen using modern molecular biology tools. Never in the history of humankind has a virus been genetically sequenced so intensively during a pandemic, with repositories currently including more than 5.3 million SARS-CoV-2 sequences. While this generates massive scientific knowledge (as tracked by PubMed trends) and opportunities for developing therapeutic and preventive strategies, it also generates a lot of anxiety in the general public.

Many different phylogenies have been developed to date to account for SARS-CoV-2 evolution, using different terminology (e.g., lineage, clade and variant). The most commonly used in the technical literature so far have been the ones generated by GISAID (www.gisaid.org), NextStrain (https://nextstrain.org), PANGOLIN (https://pangolin.cog-uk.io) and Public Health England, but since May 2021 the WHO introduced a simplified nomenclature based on the Greek alphabet, focusing on variants of concern (VOC), variants of interest and variants under monitoring.

For 2 years, plenty of variants (more than 600 according to PANGOLIN phylogeny) have spread across the planet and filled journal headlines. Finally, the current SARS-CoV-2 landscape sees a major VOC largely dominating over previous sublineages. Such variant was originally named B.1.617.2 by PANGOLIN phylogeny, 21A/S:478K by NextStrain, VUI-21APR-02 by Public Health England, G/452R.V3 by GISAID and finally referred to as ‘Delta’ VOC in the simplified WHO nomenclature. Since April 2021, Delta VOC suddenly and massively replaced the former Alpha VOC and is currently leaving marginal space to any other sublineages (including Mu, Beta and Gamma). This predominance was largely achieved thanks to a set of shared immune escape mutations in the Spike protein receptor-binding domain (L452R, T478K and P681R) that conferred better viral fitness (i.e., better replication and transmissibility) than Alpha and hence a higher reproduction number. While those shared mutations represent the backbone of Delta, many more mutations have been accumulating in time, creating derivative Delta sublineages sometimes referred to by the media as ‘Delta Plus’. These sublineages have grown in numbers and identified with the ‘AY’ alias by PANGOLIN, counting 130 members (plus 79 more submembers) as of 12 January 2021.

While many of these variants are only defined by synonymous (silent) nucleotide changes and hence have no structural differences, several of them are instead defined by nonsynonymous amino acid mutations in one or more relevant viral proteins. Of particular importance are amino acid mutations within the Spike protein, which is to date the only correlate of immunity and hence the target of neutralizing antibody-based therapeutics (either monoclonal antibodies or convalescent plasma) and preventive vaccines.

Supplementary Table 1 summarizes the 43 AY sublineages (out of 209) harboring Spike mutations, with details on the expected impact of the five sublineages harboring mutations within the Spike receptor-binding domain of subunit S1 (i.e., amino acids 319–541).

Among them, AY.49 has a very interesting mutation, termed Q493E, which has been associated with resistance to both bamlanivimab and etesevimab mAbs [1–3]. The Q493R mutation emerged during treatment with this cocktail [4–10], while Q493K has emerged in a single patient after treatment with casirivimab plus imdevimab [11,12]. Q493 mutations are exceedingly rare in other SARS-CoV-2 isolates, suggesting selective pressure rather than spontaneous convergent evolution. Similarly, Q493K is one of the founding mutations of the novel Omicron VOC from South Africa (a.k.a. B.1.1.529 or BA.1/BA.2 in PANGOLIN, 21K/21L/21M in NextStrain, GH/484A in GISAID, or VUI-2021-NOV01 in Public Health England).

Of interest, S255F which occurs in AY.106 was also recovered from an immunocompromised host in the absence of neutralizing antibody-based therapeutics [13], suggesting within-host evolution.

While Delta has clearly outcompeted Alpha and Gamma [14], Delta is unlikely to be the ultimate SARS-CoV-2 strain. Reports from South Africa, Scotland and Denmark suggest that the Omicron VOC is more transmissive and able to rapidly misplace Delta [15]: whether lockdown and travel restriction will be able to confine Omicron remains unknown, but experience with the former VOCs suggests containment is likely to be futile, especially for a variant that has a higher basic reproduction number. Supplementary Figure 1 shows how the *R*_0_ of SARS-CoV-2 has grown from the original Wuhan strain (2.4–3.4) to Alpha (4–5) to Delta (5–8), and how there is still theoretical room for getting values as high as for measles virus (12–18). It can also be expected that with massive circulation Omicron will split into many different sublineages, as it has happened with former VOCs Alpha (eight sublineages), Beta (five sublineages), Gamma (20 sublineages) and Delta (125 sublineages). Even when vaccines inducing sterilizing immunity would become available, the herd immunity threshold could be exceedingly high to be achieved if the elicited immunity is shortlasting.

One of the major lessons from the first 2 years of the pandemic is that in westernized societies modern medicine has saved the lives of millions of individuals at the price of compromised immunity and produced a population of immunocompromised patients who provide vulnerable hosts where SARS-CoV-2 can replicate for prolonged periods of time, resulting in within-host variation. This is an unprecedented landscape for pandemics, which could alter the trajectory of natural evolution. Repeated emergence of Spike variants largely deviating from the originator lineage can hence be anticipated for the coming years, as previously happened for VOCs (Alpha [23 mutations [16]] and Omicron [32 mutations [15]]) or lineages that poorly propagated (e.g., A.VOI.V2 [11 Spike mutations [17]] B.1.1.318 [14 mutations [18]] and B.1.616 [ten mutations [19]]), making clear that the number of Spike mutations *per se* is not a predictor of SARS-CoV-2 fitness.

With mounting evidence suggesting that vaccine efficacy declines significantly after as few as 6 months from the second dose, and considering that sterilizing immunity is not achievable with the current generation of nonmucosal vaccines (leaving room for transmission by vaccines and preventing herd immunity to the immunocompromised nonresponders), sudden and unpredictable Spike evolution calls for urgent research into new different types of vaccines. While it is unclear to date whether whole virus vaccines would provide better correlates of immunity, this is a road that is worth being explored in addition to Spike-only-based vaccines.

## Future perspective

Although, the early successes with vaccines and antibody-based therapies in the 1st year of the pandemic gave rise to a premature optimism that the pandemic could be contained in the foreseeable future, it is now clear that humanity might be in for a long struggle with SARS-CoV-2, given its remarkable ability to evolve via single mutations, insertions, deletions or recombination. This is difficult news for COVID-19-weary populations of the world who are entering their 3rd year of the pandemic. However, we believe that public health authorities need to have a frank discussion with the public, admitting the likelihood of the protracted struggle that will see successes, reverses, continued disruption to the norms of everyday life and suffering for many in the form of disease and death. The public in turn needs to be reassured that the way out of this calamity is by continued investment in basic science, clinical research and public health, since the road to normality with an endemic SARS-CoV-2 requires better vaccines and more effective therapies to prevent infection and disease and reduce morbidity and mortality, respectively.
